# Immunosuppression *via* Tenascin-C

**DOI:** 10.18632/oncoscience.210

**Published:** 2015-08-21

**Authors:** Matteo Bellone, Sara Caputo, Elena Jachetti

**Affiliations:** Division of Immunology, Transplantation and Infectious Diseases, Cellular Immunology Unit, IRCCS San Raffaele Scientific Institute, Milan, Italy

**Keywords:** prostate cancer, metastasis, cancer stem cell, tenascin-c, immunosuppression

Metastasis accounts for most of the prostate cancer-related deaths, and the presence of more than two metastatic lymph nodes at radical prostatectomy and extended pelvic lymph node (PLN) dissection is an independent predictor of prostate cancer specific mortality [1], thus suggesting that PLN invasion is an important event in the history of the disease. However, despite its clinical significance, little is known about the mechanisms favoring dissemination and survival of cancer cells at sites of future metastasis. This is particularly intriguing for cancer cells invading lymph nodes, where both innate and adaptive immunity should rapidly recognize and eliminate disseminated cells.

Another biological conundrum is when in the history of prostate cancer, a neoplastic cell detaches from the primary lesion and seeds the metastatic niche. Indeed, even patients affected by less aggressive prostate cancer may harbor occult lymph node metastasis, which might remain quiescent and shielded from immune surveillance for years before growing and becoming clinically relevant.

Quiescence and immune evasion are characteristics of stem cells, and cancer cells with stem cell properties have been implicated in early metastatic dissemination [2]. Thus, we hypothesized that cancer stem-like cells (CSC) both at primary and metastatic sites possess mechanisms of immune evasion. We investigated this biological issue in transgenic adenocarcinoma of the mouse prostate (TRAMP) mice that express the SV40 early genes (large and small T antigens; Tag) under the control of the probasin regulatory element selectively in the prostate epithelium. Indeed, androgen-promoted expression of Tag at puberty leads to prostate intraepithelial neoplasia (PIN; week 6-12) that invariably progresses to adenocarcinoma (week 12-18). PLN metastases from adenocarcinoma are infrequent in TRAMP mice, and in our cohort of more than 100 mice they occurred after week 17.

Utilizing the sphere assay, we established long-term prostate CSC lines from unsorted prostate cells obtained from different stages of TRAMP progression [3]. CSC were endowed with self-renewal, multipotency, and tumorigenicity capacities. Transcriptome analysis showed that genes upregulated in each stage-specific prostate CSC were significantly associated with distinct clinical subgroups of prostate cancer patients, thus indicating that mouse CSC may define human prostate cancer progression signatures [3]. Although CSC obtained from PIN lesions (hereafter named TPIN-SC) were killed *in vitro* by NK cells and cytotoxic T lymphocytes, they generated tumors when injected in immunocompetent mice [4], thus suggesting they possess mechanisms of immune evasion.

Curiously, CSC phenotypically and functionally identical to TPIN-SC were obtained from histopathologically negative PLN (hereafter named TLN-SC) of TRAMP mice at about 10-12 weeks of age [5], thus demonstrating that lymph node invasion can already occur at the stage of PIN in TRAMP mice. Concomitantly, at that stage TRAMP mice develop full cytotoxic T cell tolerance to Tag [6], which behaves in this model as a tissue-restricted tumor associated antigen.

**Figure 1:**
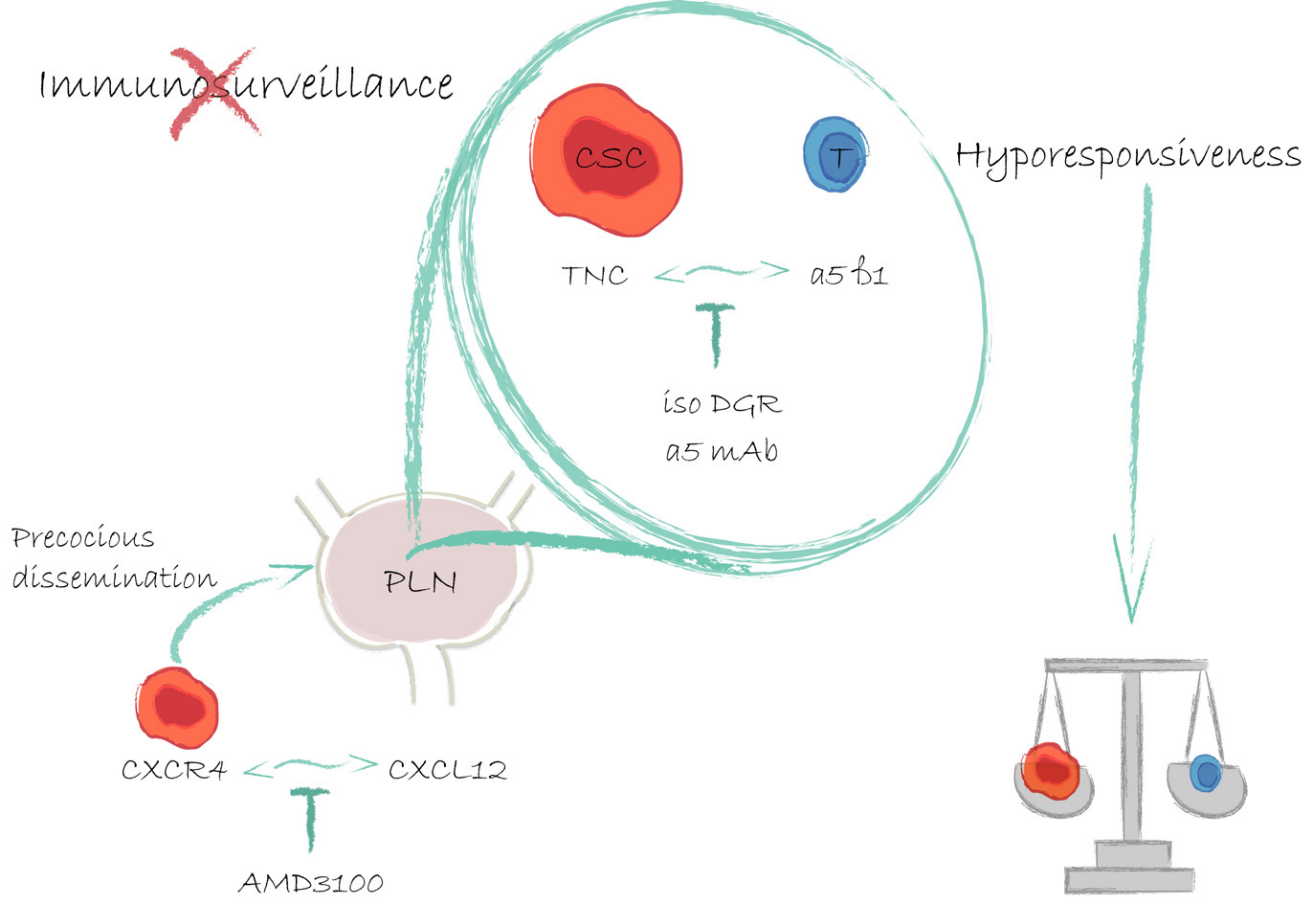
Prostate cancer stem-like cells early migrate to prostate draining lymph nodes through the CXCR4-CXCL12 axis, and dampen T cell responses with Tenascin-C Already at the stage of PIN, prostate CSC are attracted to PLN by a CXCL12 gradient. Both at the primary site and in the metastatic niche CSC use TNC to overcome immune surveillance. Drugs that either block the CXCR4-CXCL12 or the TNC-α5β1 axes could reduce metastasis occurrence.

Both *in vitro* and *in vivo* TPIN-SC and TLN-SC, and not CSC obtained from frank prostate tumors, used Tenascin-C (TNC), an extracellular matrix protein of stem cell niches [7], to interact with α5β1 integrin on the cell surface of both human and murine T cells, thus inhibiting T cell receptor-dependent activation, proliferation and cytokine production [5]. Moreover, we found that TNC was expressed in the extracellular matrix and in the acinar basement membrane in both normal human and TRAMP prostate and low-grade PIN, its expression increased in high-grade PIN, returned to basal levels in prostate adenocarcinoma, and it was again overexpressed in lymph node metastases [5]. All together, these findings strongly support the role of TNC in the early phases of disease progression, likely fostering generation of the metastatic niche [7], and protecting disseminated CSC from immune surveillance. Thus, targeting TNC at the stage of high-grade PIN, and in low-risk prostate cancer patients (i.e., those with Gleason scores ≤ 6, PSA concentrations <10 ng/mL, or T1-T2a; Figure 1) might prevent metastasis occurrence.

TPIN-SC and TLN-SC also expressed CXCR4 and migrated in response to its ligand CXCL12, which was overexpressed in PLN upon PIN development. Interestingly, *in vivo* administration of AMD-3100, a CXCR4 inhibitor, prevented establishment in PLN of an immunosuppressive microenvironment [Figure 1; ref. 5], suggesting this as an alternative strategy to prevent CSC dissemination and metastasis.

Although a recent study also involving low-risk prostate cancer patients confirmed a significant reduction in the rate of death and the risk of metastases in the radical prostatectomy group as compared with the watchful-waiting group [8], overtreatment remains high, and still debated is who among low-risk patients will benefit most from radical prostatectomy. Based on what we have found in the TRAMP model and in human samples [5], we propose that besides DNA signatures, criteria of local immune suppression (e.g., expression of TNC) and precocious PLN dissemination (e.g., expression of CXCL12 and/or TNC) should be investigated as predictors of prostate cancer specific mortality.
